# Key statistical recommendations for medical journals: insights from the Global Burden of Disease Study Collaborators

**DOI:** 10.7150/ijms.119771

**Published:** 2026-03-04

**Authors:** Michal Ordak

**Affiliations:** Department of Pharmacotherapy and Pharmaceutical Care, Faculty of Pharmacy, Medical University of Warsaw, Banacha 1 Str., 02-097 Warsaw, Poland.

**Keywords:** biostatistics, medical journals, statistical reviews, surveys and questionnaires

## Abstract

**Background:**

Biostatistics is essential in personalized medicine, enabling the analysis of complex data, optimizing treatment strategies, and ensuring robust clinical trial designs for patient-specific therapies. The aim of the article was to find out the opinion of Global Burden of Disease Collaborators on the statistical recommendations that should be implemented in medical journals.

**Materials and Methods:**

The study involved 150 GBD Collaborators who authored articles between 2018 and 2023 under the research coordination of the Institute for Health Metrics and Evaluation. The analysis included 11 statistical recommendations and parameters such as the Hirsch index, number of published articles, and scientific seniority. Additionally, opinions were assessed regarding the percentage of accepted scientific manuscripts that meet statistical validity.

**Results:**

The key recommendation highlighted by the GBD collaborators is to ensure regular statistical reviews when there is uncertainty about the quality of the authors' analyses (p < 0.001). The remaining recommended guidelines primarily involve the publication of statistical recommendations (50%) and their inclusion on journal websites (53%). The GBD Collaborators, who assert that a lower percentage of accepted articles in medical journals are statistically correct, recommend that authors consult the statistical recommendations posted on journal websites before submitting an article (p = 0.03) and advocate for uniform publication guidelines across journals (p = 0.01).

**Conclusion:**

More emphasis should be placed on implementing statistical recommendations in medical journals, not just publishing them.

## Introduction

Biostatistics is essential to personalized medicine, enabling the analysis of complex datasets and the design of statistically robust studies to optimize patient-specific treatments and advance clinical practice.^1^ Between 1998 and 2020, there was unfortunately no significant change in the frequency with which biomedical specialist journals carry out statistical reviews. Thirty-four percent rarely or never use this type of review.^2^ In 2005, PLOS MEDICINE pointed out that there is a growing concern that a greater proportion of published research findings are falsified.^3^ Recent literature has highlighted significant shortcomings in data analysis reporting, including incomplete methodological disclosures and a lack of transparency, which hinder the evaluation of study accuracy and reproducibility. Initiatives like those from the Equator Network emphasize the need for standardized reporting guidelines to address these critical issues across disciplines.^4^ The results of the study, published at the end of 2021, indicated that only 39% of the 2,600 accepted articles on various aspects related to COVID-19 met statistical validity. The impact factor of most of these journals was a maximum of 10.^5^ The persistence of poor quality in statistical reporting continues to be a significant issue that undermines the clarity and reliability of the information presented.^6^-^8^ Editors of medical journals may not have the expertise to fully assess the validity of the various types of statistical analyses performed by the authors.^9^ Another issue concerns the challenge editors face when determining how to select statistical reviewers in cases where there are doubts about the quality of the analysis conducted by the authors.^10^-^11^ For this reason, there has been a slow start to the publication of statistical recommendations in specific journals in recent years. However, as highlighted by the authors in the article published in PLOS ONE, both before and after the publication of editorial guidance, instances of poor statistical reporting persist.^12^ To improve the quality of published research in medical journals, additional statistical recommendations should be implemented. However, simply publishing these recommendations does not guarantee that authors will follow them, as issues persist with editors struggling to find statistical reviewers and articles being published with flawed statistical analyses. Leading journals such as The Lancet and JAMA, for example, use statistical reviews.^13^,^14^ The international Global Burden of Disease project conducts significant research on global disease estimates and related health risk factors. The Institute for Health Metrics and Evaluation (IHME) is the research institute responsible for coordinating all work related to this project. The results of ongoing GBD studies are published in a number of journals such as The Lancet and JAMA, among others. The GBD project leverages advanced statistical methods to analyze global health, supported by a diverse network of experienced collaborators in public health, medicine, epidemiology, and statistics.^15^ As mentioned, previous research has been based on understanding the opinions of members of the editorial boards of medical journals.^2^ There is a significant lack of input from Global Burden of Disease (GBD) project collaborators, whose expertise in statistical recommendations could provide valuable insights. As researchers frequently publishing in prestigious journals like The Lancet, their perspectives could enhance awareness of the need for implementing statistical recommendations, complementing editors' views and highlighting the importance of diverse expert opinions in medical research. In this study, a series of additional statistical recommendations were proposed, and the experts' opinion regarding the percentage of accepted manuscripts that are statistically sound was also considered. Understanding the viewpoints of GBD collaborators provides a unique perspective on the researched topic, distinct from that of the editors. For this reason, GBD collaborators were included to carry out the present study, the results of which are presented in this manuscript. Getting to know experts who are scientifically active in various fields of medicine on recommended statistical recommendations is the first study of its kind to be conducted. The primary aim of this study was to investigate the perspectives of Global Burden of Disease (GBD) contributors regarding the implementation of various statistical recommendations in the routine practices of medical journals. Specifically, it aimed to assess the perceived value and practicality of integrating these recommendations into editorial processes. Additionally, the study aimed to evaluate GBD collaborators' assessments of the statistical validity of accepted manuscripts, aiming to determine the proportion meeting established criteria. Another objective was to establish relationships between the research findings and the scientific contributions of the expert group under study.

## Materials and Methods

### Study group

The responses were obtained from 150 out of 450 (33%) surveyed GBD Collaborators. This is a similar percentage of survey response rates observed in other similarly conducted survey studies. For instance, research results published in PLOS ONE indicated that out of 364 surveyed journals, the response rate was 28%.^2^ The study involved 150 GBD Collaborators from all continents who published articles between 2018 and 2023 as part of research coordinated by the Institute for Health Metrics and Evaluation (IHME). The sample size of 150 GBD Collaborators was determined to capture a diverse range of academic experiences and publication histories in GBD-related research across different journals. This approach was based on the need to capture a diverse spectrum of contributors within the Institute for Health Metrics and Evaluation database, encompassing authors who have published GBD articles from 2018 to 2023. This selection criterion aimed to reflect the broad range of scientific contributions and expertise present within the global GBD research community, enhancing the comprehensiveness and validity of the study's findings. The survey was sent to the email addresses of corresponding authors and other co-authors of published GBD articles. The first wave of emails was sent on July 1st, 2023, and data collection was finalized on August 31st, 2023. Co-authors were contacted by clicking on their names on the page of the published article for subsequent redirection to their respective PubMed pages containing all their articles. Subsequently, email addresses of GBD collaborators were extracted from these articles. Prior to survey distribution, inclusion criteria for participation in the study were based on authors and co-authors of GBD articles published between 2018 and 2023. These criteria ensured that all participants had direct involvement in GBD research during the specified period. The identification of each GBD collaborator was verified by confirming the scientific unit indicated in the GBD article. Additionally, each participant responded to inquiries, including questions about the Impact Factor of the journal in which their GBD article was published. To ensure data completeness and reliability, participants who did not fully complete the survey were excluded from the final analysis. This criterion was applied rigorously to maintain the integrity of the study's findings. The study was conducted with approval from the Bioethics Committee of the Warsaw Medical University (approval number AKBE No. 48/2024). The survey was completed conscientiously. Before completing the survey, each GBD collaborator was informed about the purpose of the analyses and the data collection for this study. They were also given the right to discontinue participation and possibly exclude their responses. By completing the survey after understanding the research objectives, participants gave informed consent to participate in the study. Measures were implemented to prevent identification of individual respondents. The survey specified that the data controller was solely the author of this article, along with their university affiliation and professional address.

### Survey

The survey was developed and hosted on the 'Webankieta' platform and distributed via e-mail. The survey is included in the first appendix (Appendix 1). The survey was developed based on the author's extensive experience derived from collaborating with members of the editorial boards of medical journals on conducting statistical reviews. The statistical recommendations included in the survey also stem from the latest published data concerning issues related to statistical review practices among members of the World Association of Medical Editors (WAME).^16^ Such recommendations were presented in June 2024 at the 8th World Conference on Research Integrity.^17^ However, opinions regarding them have not yet been analyzed, hence their incorporation into the survey. It included questions on the gender, age and continent of origin of GBD contributors. Further questions included seniority as a researcher, Hirsch coefficient and number of published articles in medical journals, including as co-author. In the 2023 publication in the Pakistan Journal of Medical Sciences, Shah and Jawaid indicated that the Hirsch index could be utilized for comparing scientists in the context of scholarships and grants, as well as for predicting future achievements of researchers. As highlighted by these authors, the h-index is increasingly recognized and widely used in the scientific community, with significant implications for assessing the scholarly contribution of academic faculty members and the standards of medical journals.^18^ Despite the controversy surrounding the Hirsch Index, its specified advantages have led to its inclusion in the survey. Another question pertained to the Impact Factor of the journal in which the GBD project results were published, understood as the Impact Factor displayed on the journal's official website, providing additional context about the publication profiles of the survey respondents. Another important question assessed the respondents' subjective opinions on what percentage of accepted manuscripts in medical journals they believed met statistical validity, defined as analyses conducted in accordance with recognized statistical standards and practices. The term 'accepted articles' refers to manuscripts that have been accepted for publication in medical journals but are not necessarily published yet. At the very end of the survey, 11 statistical recommendations were included for GBD contributors to identify those they felt were important to implement in medical journals. They have been extracted from previously published recommendations in this area so that, for the first time, it was possible to get an expert opinion on them from GBD collaborators. To improve the quality and reliability of statistical analyses reported in medical journals, several key recommendations have been proposed.

### Statistical analysis

Statistical analysis was carried out with the SPSS25 statistical software (IBM SPSS Statistics, Armonk, NY: IBM Corp.). In order to test whether there was a statistically significant difference in the proportion of GBD colleagues selecting each specific recommendation (e.g., regular statistical reviews), a chi-square test was used to compare the observed proportion for each recommendation (i.e., "yes" or "no") with an expected neutral proportion of 50%, which assumes no preference for or against the recommendation. This test determined whether a statistically significant majority of contributors selected each specific recommendation. For other analyses involving categorical variables with more than two categories (e.g., research internship, Hirsch index), a chi-square test was used to examine statistically significant associations with the recommendations. The strength of these associations was measured using Cramér's V coefficient, which is appropriate for multi-categorical variables. A 95% confidence interval was calculated for the Cramér's V effect size, with values of ≤ 0.2 interpreted as weak, 0.2 < V ≤ 0.6 as moderate, and > 0.6 as strong, consistent with conventional interpretations of Cramér's V. The assessment involved counting the number of experts (n) and calculating the percentage (%) of responses to various survey questions. Using Spearman correlation analysis, it was tested whether there was a statistically significant correlation between the age of GBD collaborators and their opinion on what percentage of accepted manuscripts were statistically correct. A p value <0.05 was taken as the statistically significant level.

## Results

### Study group

150 GBD Collaborators took part in the study, the largest proportion of whom were Asian and male. The Hirsch index of most GBD contributors ranged between 11 and 50. The seniority of 41.3% of them was up to 10 years, while a slightly smaller proportion, 36%, had between 11 and 20 years. The number of published articles in the largest proportion of GBD contributors was above 100. The Impact Factor of the journal in which the GBD results were published was identified by the majority of respondents as higher than 100 (Table 1). This is explained by the exceptionally high Impact Factors of several leading medical journals during the COVID-19 period, including The Lancet, which temporarily exceeded this value. The median age was 41 years (range: 23-79).

### Statistical recommendations in medical journals

The presented data in Table 2 illustrate the varying levels of support among GBD collaborators for eleven statistical recommendations aimed at medical journals. Notably, a statistically significant majority of contributors (67%) advocated for regular statistical reviews in instances of analytical uncertainty. This was determined by applying a chi-square goodness-of-fit test to compare the observed proportion of advocates (67%) with an expected neutral proportion of 50%, which assumes no preference for or against the recommendation χ²(1) = 18.03; p < 0.001. This finding underscores the potential impact of enhancing statistical rigor in manuscript evaluation processes. Moreover, insights revealing preferences for including these recommendations on journal websites (53%) and advocating for uniform guidelines across medical publications (45%) highlight the ongoing discourse on standardizing statistical practices (Table 2).

The median number of recommendations selected was 4 among which there were mainly regular statistical reviews when there were doubts about the quality of the analysis performed and regular publication of statistical recommendations.

### Percentage of accepted articles meeting statistical validity in medical journals

**Figure 1:**
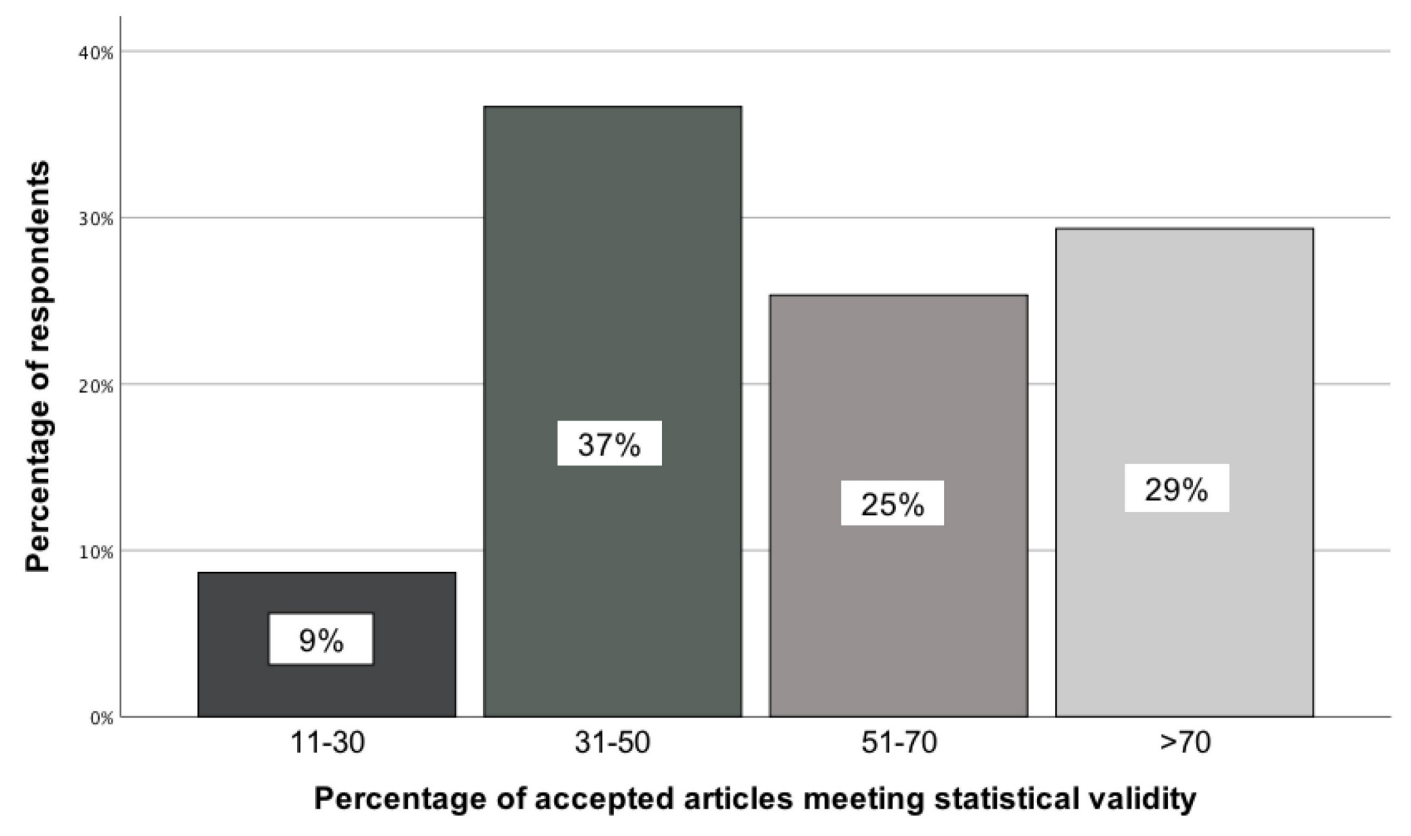
Opinion of GBD collaborators on the percentage of accepted articles meeting statistical validity [11-30; 31-50; 51-70; >70].

GBD collaborators expressed divergent views on what percentage of accepted scientific manuscripts in medical journals met statistical validity. This was determined by comparing the observed distribution of responses across four categories using a chi-square goodness-of-fit test, χ²(3) = 25.31; p < 0.001. Just under 40 percent of GBD colleagues indicated that this percentage ranged from 31% to 50% (Figure 1).

The viewpoint of GBD collaborators regarding the percentage of accepted manuscripts with correctly performed statistical analyses does not demonstrate a statistically significant association with the Hirsch index, scientific seniority, or the number of articles published by collaborators in GBD medical journals (p > 0.05). Similarly, no significant correlation was found with their age (r_s_ = 0.07; p = 0.41). This analysis aimed to examine whether the Hirsch index, scientific seniority, or publication experience were associated with opinions on the statistical validity of accepted manuscripts. The percentage of accepted articles meeting statistical validity was assessed across four categories: (1) 11-30%, (2) 31-50%, (3) 51-70%, and (4) >70%. The Hirsch index was divided into three categories: (1) 1-10, (2) 11-50, and (3) 51+. Research internship was categorized as: (1) 1-10 years, (2) 11-20 years, (3) 21-30 years, and (4) >30 years. The number of articles published was divided into three categories: (1) 1-50, (2) 51-100, and (3) >100 articles. The table below shows the respondents' view of the study percentage according to their Hirsch index, academic seniority, and number of published articles in medical journals. The Hirsch index was narrowed down to three groups due to the fact that only a few GBD contributors had more than 100. The same applies to the number of published articles in medical journals. In the group of GBD collaborators with a Hirsch index of 1 to 10, 38% believe that the percentage of accepted manuscripts in which the analysis is correctly performed ranges from 31 to 50. Similarly, in the group with a Hirsch index of 11 to 50, this proportion is 39. Only slightly more people with a Hirsch coefficient of more than 50 indicated this percentage to be above 70. A similar interpretation applies to the number of published articles in medical journals. In the groups of GBD collaborators divided by their academic seniority, this percentage was also identified by most of them as 31-50. Respondents with a higher number of publications (>100) were more likely to believe that more than 70% of accepted manuscripts in medical journals meet statistical validity (41%) compared to those with fewer than 51 publications (19%), suggesting a potential link between publication experience and confidence in statistical practices (Table 3).

**Figure 2:**
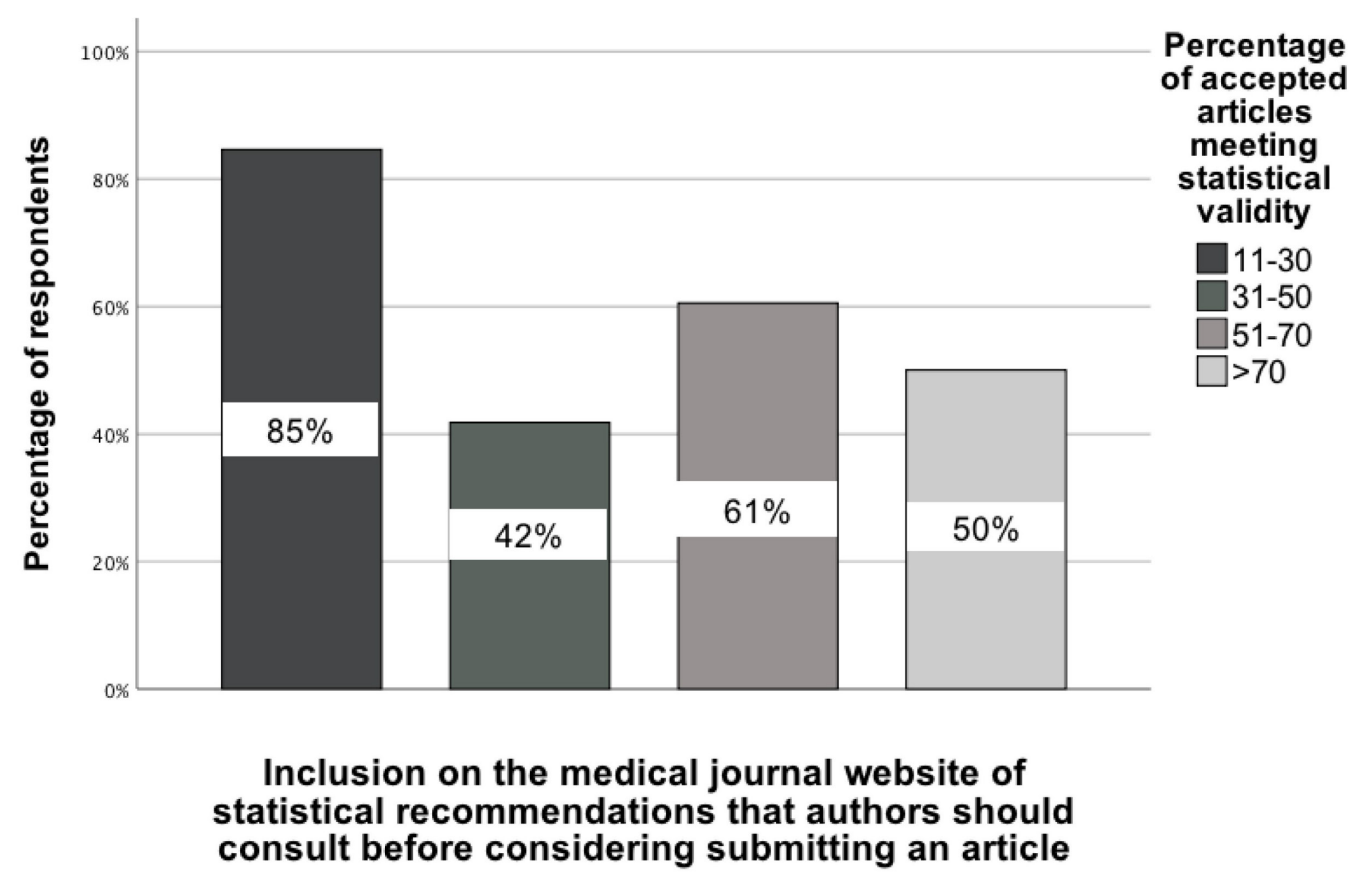
The association between GBD colleagues' opinion of the percentage of accepted manuscripts in medical journals and their indication of a recommendation to post statistical recommendations on the website for authors to read before they submit [0-100%].

The statistical analysis indicated two significant and practically important associations regarding the opinion of GBD colleagues on the percentage of accepted articles in medical journals in which the statistical analysis was correctly performed. The first pertains to whether authors intending to submit an article to a particular journal read the statistical recommendations posted on the journal's website, as determined by the association between selecting this recommendation and responses to the four-category question on statistical validity, χ²(3) = 8.99; p = 0.03; Vcr = 0.25 [95% CI: 0.21-0.28]. For this analysis, the four categories in the statistical validity question were: (1) 11-30%, (2) 31-50%, (3) 51-70%, and (4) >70%. In the group of GBD collaborators who believe that only 11-30% of accepted manuscripts meet statistical validity, up to 85% of them indicated that such a recommendation should be implemented. A smaller percentage of other GBD collaborators indicated this type of statistical recommendation (Figure 2).

**Figure 3:**
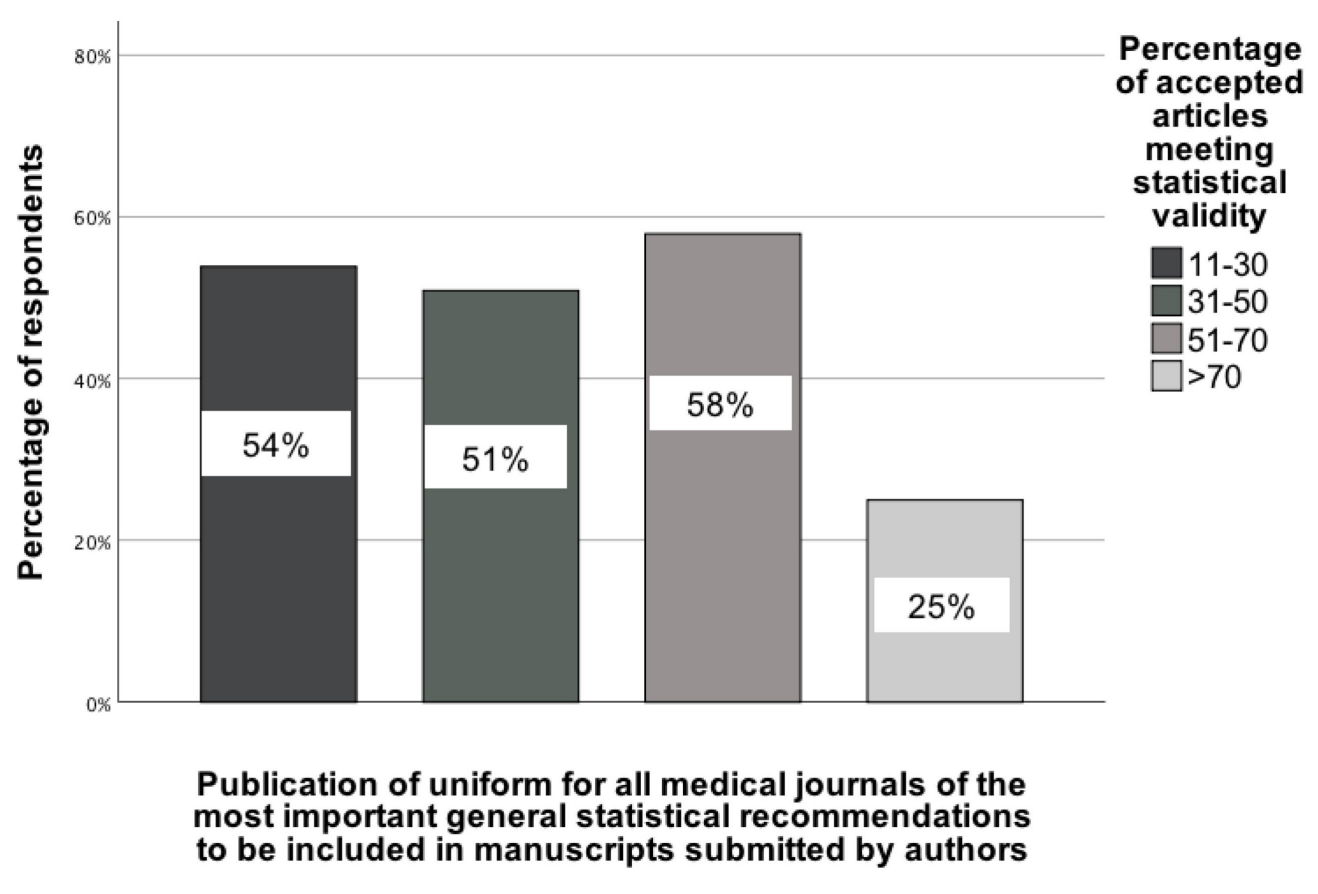
The association between the opinion of GBD colleagues on the percentage of accepted manuscripts in medical journals and their indication of a recommendation to publish uniform statistical recommendations for all medical journals [0-100%].

The second association pertains to the recommendation to publish uniform statistical guidelines for all medical journals, as determined by examining the association between selecting this recommendation and responses to the four-category question on statistical validity, χ²(3) = 10.83; p = 0.01; Vcr = 0.27 [95% CI: 0.19-0.35]. For this analysis, the four categories in the statistical validity question were: (1) 11-30%, (2) 31-50%, (3) 51-70%, and (4) >70%. In the group of GBD collaborators who believe that more than 70% of accepted manuscripts meet statistical validity, only 25% of them indicated that statistical guidelines should be implemented for all medical journals. For the remaining GBD collaborators, the percentage indicating support for this recommendation was higher (Figure 3).

### Statistical recommendations and scientific parameters of GBD collaborators

**Figure 4:**
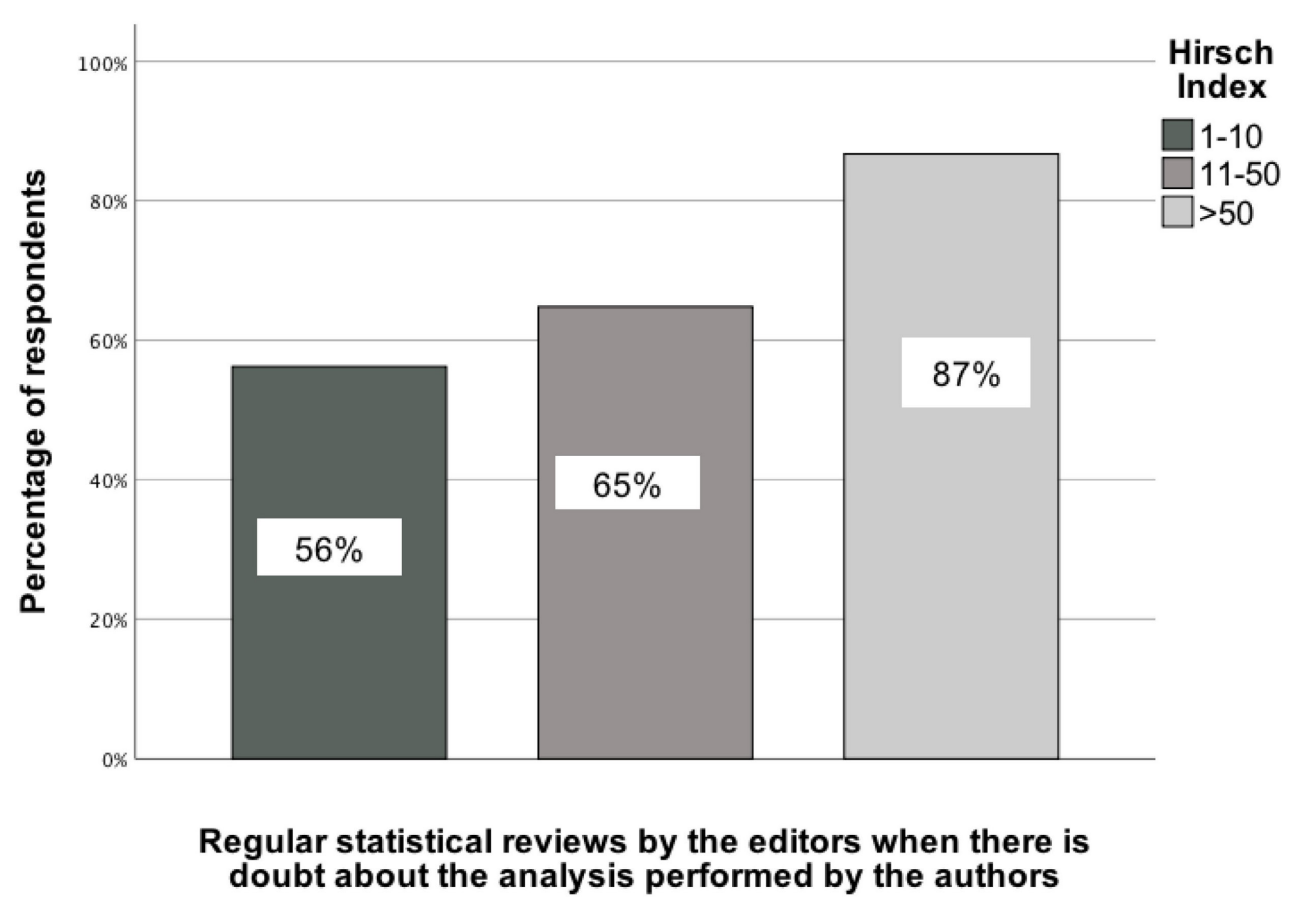
The association between the Hirsch index of GBD contributors and their indication of a recommendation to carry out regular statistical reviews when editors have doubts about the quality of the analysis performed by the authors [0-100%].

The analyses conducted revealed several statistically significant associations between the scientific parameters of GBD collaborators and statistical recommendations. The first association pertained to the Hirsch index of GBD contributors and their selection of the recommendation to regularly conduct statistical reviews when editors have doubts about the quality of the analysis performed by the authors. This association was determined by comparing responses across three Hirsch index categories: (1) 1-10, (2) 11-50, and (3) 51+. χ²(2) = 7.15; p = 0.03; Vcr = 0.22 [95% CI: 0.12-0.32]. In the group of GBD colleagues with the highest Hirsch index, just under 90% of them chose this type of recommendation, while in the group with the lowest Hirsch index, only over 56% did so (Figure 4).

**Figure 5:**
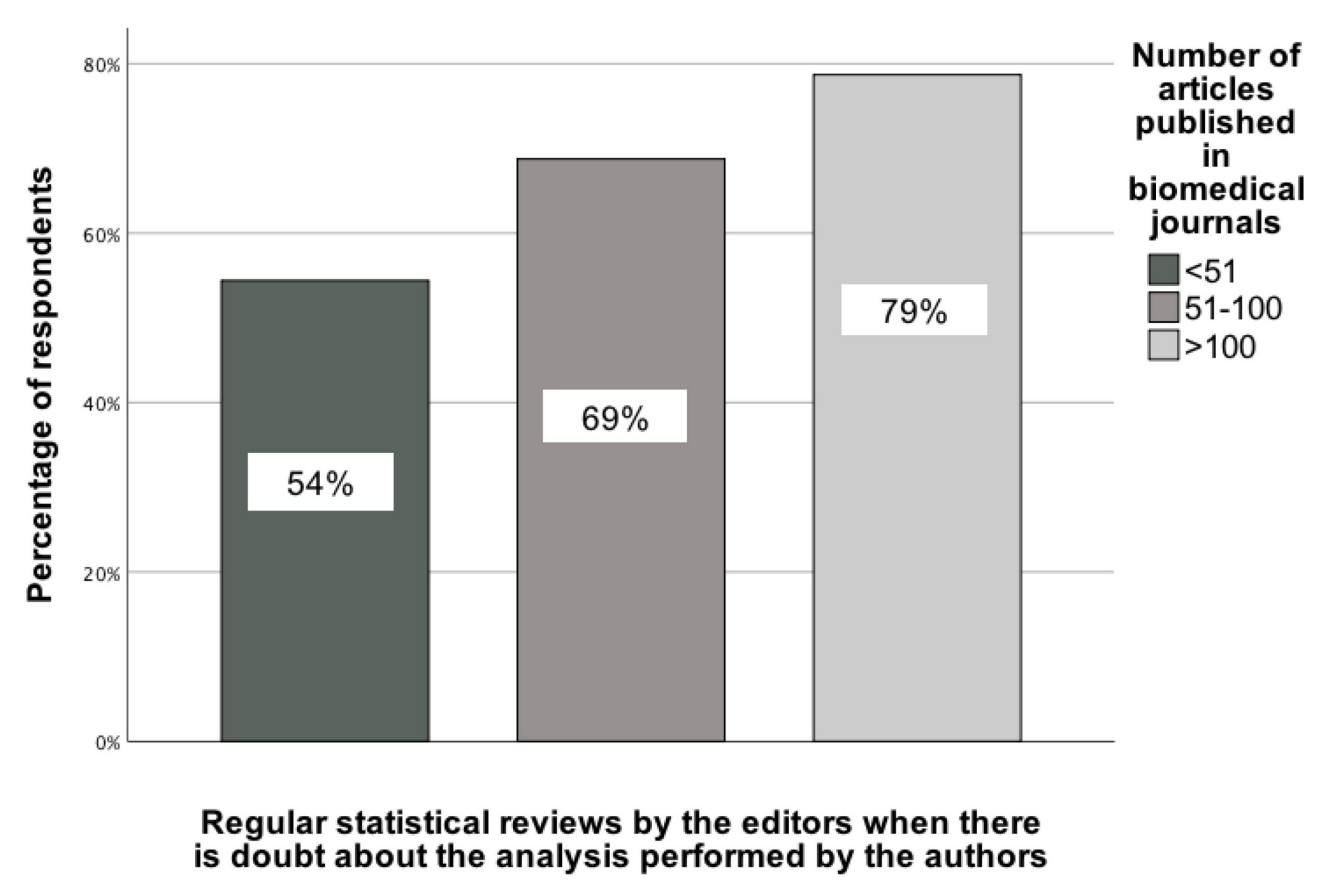
The association between the number of GBD fellows' published articles in medical journals and their indication of the recommendation that statistical reviews should be carried out regularly when editors have doubts about the quality of the analysis performed by the authors [0-100%].

A similar interpretation applies to the number of articles published in medical journals, where responses were compared across three categories: (1) 1-50 articles, (2) 51-100 articles, and (3) 101+ articles. χ²(2) = 7.95; p = 0.02; Vcr = 0.23 [95% CI: 0.003-0.46] (Figure 5).

**Figure 6:**
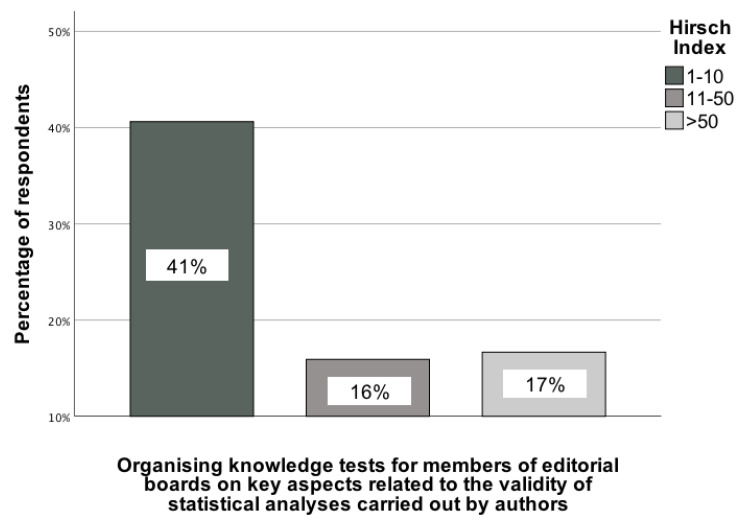
The association between the Hirsch index of GBD contributors and their indication of a recommendation indicating that knowledge tests should be conducted for editorial board members of medical journals on basic aspects related to the statistical analysis performed by the authors [0-100%].

An interesting statistically significant association was observed between the Hirsch index (categorized as 1-10, 11-50, and 51+) and the opinion of GBD collaborators that knowledge tests should be conducted for editorial board members of medical journals on basic aspects related to the statistical analysis performed by the authors. This association was determined by comparing responses across these categories, χ²(2) = 9.03; p = 0.01; Vcr = 0.25 [95% CI: 0.02-0.47] (Figure 6). The recommendation to implement knowledge tests for editorial board members was mainly selected by GBD collaborators with the lowest Hirsch index (1-10), with 41% of them supporting its implementation.

**Figure 7:**
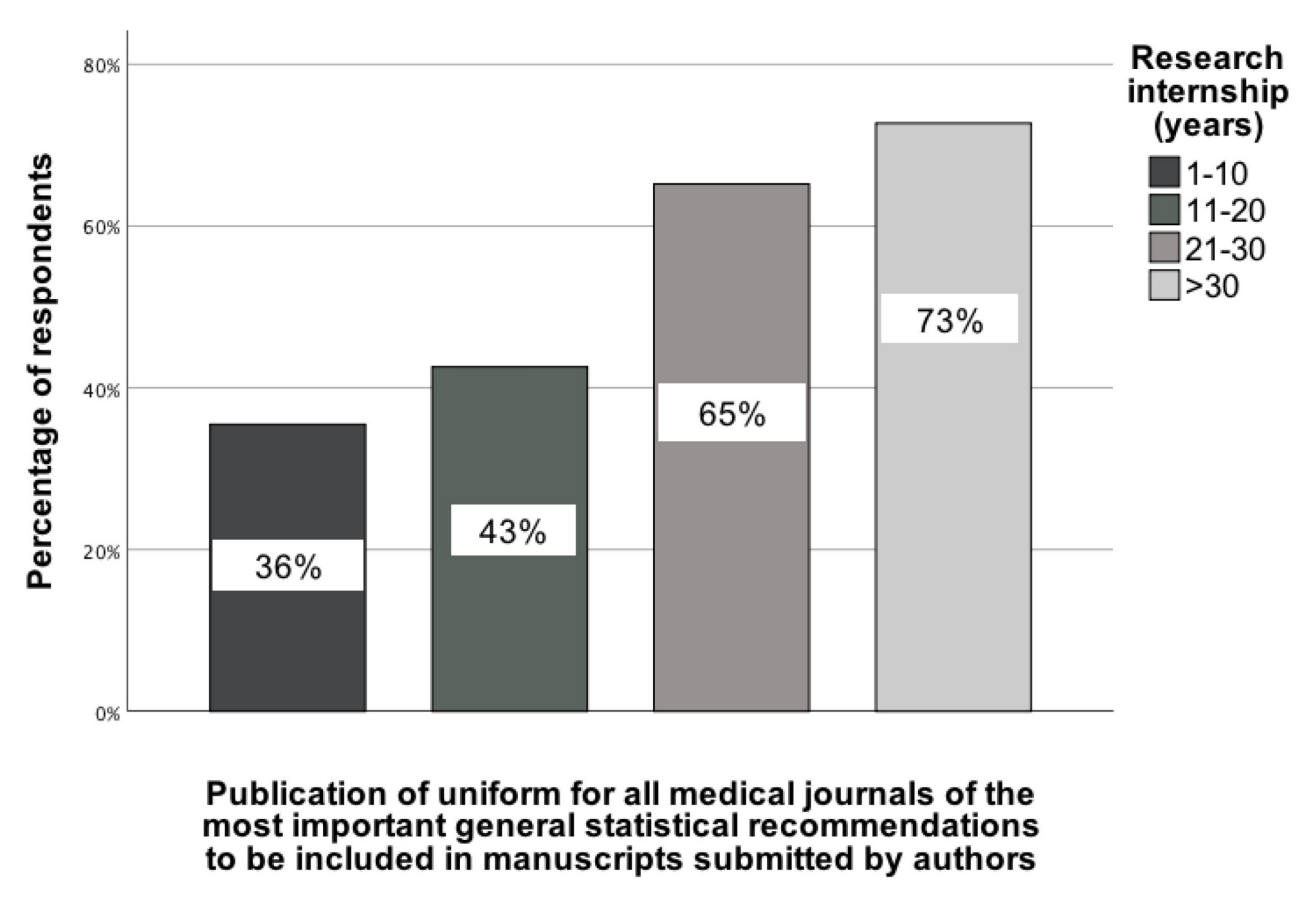
The association between the seniority of GBD contributors and their indication of a recommendation indicating that there should be uniform statistical recommendations that authors should consult before submitting an article to a medical journal [0-100%].

Another statistically significant association was observed between seniority in science (research internship, categorized as 1-10 years, 11-20 years, 21-30 years, and >30 years) and the recommendation to create uniform statistical guidelines that authors should be familiar with before submitting an article to a medical journal. This association was determined by comparing responses across these categories, χ²(3) = 9.59; p = 0.02; Vcr = 0.25 [95% CI: 0.03-0.5] (Figure 7). Notably, this recommendation was selected primarily by those with the highest level of research internship (>30 years), with 73% of them indicating support for the implementation of uniform statistical guidelines.

### Other statistical recommendations

The rest of the recommendations identified by the GBD collaborators comprise individual statements. The receipt by editors and statistical reviewers of remuneration for their specialised reviews is indicated. One of the notable recommendations is to include a biostatistician or methodologist as a co-author for manuscripts involving randomized controlled clinical trials (RCTs), cohort studies, and other complex designs. A key recommendation proposed by a GBD collaborator suggests that manuscripts with significant scientific innovations, yet flawed analyses, should receive guidance on how to accurately reflect research findings. An additional recommendation advocates for organizing workshops and statistical conferences specifically tailored for members of editorial boards of medical journals. According to input from two GBD contributors, there should be increased emphasis on regular recruitment of reviewers and statistical editors by members of editorial boards. Another suggestion is for journals to clearly outline on their websites the specific information authors need to include in their submissions. This can help mitigate the issue of ambiguous reviews from independent reviewers. For instance, one reviewer may endorse the correctness of the statistical analysis, while another may hold an opposing view. This type of recommendation, according to the GBD collaborator, could also make it easier for editors to deal with situations where they cannot find reviewers who can check the manuscript for the authors' statistical analysis. The last statistical recommendation reported in the open-ended question according to one GBD contributor has to do with authors including published statistical recommendations in written manuscripts such as “Statistical Analyses and Methods in the Published Literature: The SAMPL Guidelines.”^19^. In case of doubts about the quality of the analysis carried out by the authors, they could have given a concrete indication from the recommendations quoted so as to be able to specify why they presented the research results in such a way.

## Discussion

This study appears to be the first to explore expert opinions on statistical recommendations by medical journals. The choice of GBD collaborators was based on the fact that they are experts representing different fields of medicine who, through their collaboration with the IHME, publish research results on a comprehensive measure of health status. The analyses carried out are country-specific and based on data spanning many years. Gathering the opinions of this group on recommended statistical practices and using these insights to inform future implementation strategies in the daily operations of medical journals could lead to improvements in the quality of published research outcomes. The novelty of this study lies in its exploration of expert opinions on additional statistical recommendations among the surveyed group and its innovative approach to correlating these opinions with scientific achievements while also evaluating the prevalence of statistically sound manuscripts among those accepted. In other words, the study assessed whether opinions on the percentage of accepted manuscripts meeting statistical validity influenced the selection of statistical recommendations. To address these challenges and enhance the quality of statistical analyses in medical journals, several key recommendations have been proposed. Authors are encouraged to consult statistical guidelines provided on the journal's website before submitting an article and to confirm in their cover letter that they have read and adhered to these guidelines. It is also advised that authors specify in their motivation letter who is responsible for the statistical analysis of the research results, and, if significant contributions are made, this expert should be included as a co-author. Furthermore, authors are encouraged to include a certificate confirming the correctness of their analysis, such as a review by an expert biostatistician, recognizing, however, that the implementation of such a requirement may be challenging due to the limited availability of qualified experts in this field. Editors, on the other hand, are encouraged to conduct regular statistical reviews, particularly when there is doubt about the authors' analysis. Greater emphasis should be placed on recruiting qualified statistical reviewers, such as PhD students trained in biostatistics who can review manuscripts as part of their course credit. Additionally, it is recommended that medical journals regularly publish biostatistical guidelines to educate their editorial board members and that biostatistical reviewers or editors organize short online meetings to highlight common statistical errors made by authors. Professional organizations like the World Association of Medical Editors and the International Committee of Medical Journal Editors could play a pivotal role by organizing meetings to present statistical recommendations. Regular knowledge tests for editorial board members on key aspects of statistical analysis validity are also suggested, along with the publication of uniform statistical recommendations for inclusion in manuscripts submitted by authors.

The main statistical recommendation for medical journals identified by the collaborators is to carry out regular statistical reviews when editors have doubts about the quality of the analysis performed by the authors. At the moment, there is unfortunately a low quality of published research results, while the percentage of medical journals using statistical reviewers has not changed in 20 years.^2^,^8^ There should be separate and step-by-step statistical analysis (including information of software/codes) in the medical paper submitted for publication. An article published in BMC Medicine specified the statistical aspects that should be reviewed by non-statistical reviewers.^20^ However, simply publishing such recommendations is not sufficient, they should be made available to reviewers who are not statistically literate or additional recommendations should be sought. The next in order of importance identified by GBD colleagues are the publication of statistical recommendations in journals and their inclusion on the journal's website. Such recommendations aim to encourage authors to carefully review their manuscripts before submission to specific journals. An example of a journal that provides such guidance on its website is the British Dental Journal - Nature.^21^ Prestigious journals such as the New England Journal of Medicine refine and publish newer statistical recommendations using a rigorous peer-review process.^22^ Recommendations or guidelines for data analysis represent critical aspects for achieving substantial progress. However, comprehensive guidance appears to be lacking for several essential statistical topics. This gap constitutes a significant reason behind weaknesses observed in numerous analyses. Some analyses may exhibit inaccuracies stemming from insufficient statistical expertise on the part of the analyst in charge. It is recommended that medical journals publish biostatistical recommendations and include them on their website so that authors wishing to submit an article then have to read them first. Where such recommendations have not been published in a specific journal, it is recommended that statistical recommendations such as the SAMPL Guidelines recommended by the World Association of Medical Editors be posted on their website. The SAMPL recommendations could be integrated into the routine operations of medical journals.^19^ Reviewers evaluating submitted manuscripts could assess whether authors have addressed critical aspects, such as providing comprehensive descriptions of statistical methods to validate results, stating assumptions underlying statistical tests, accurately reporting p-values, and more, in accordance with the SAMPL recommendations. This serves as a guide for reviewers who may lack statistical expertise, making it easier to make decisions while writing reviews. Slightly more than 45% of GBD contributors indicated a recommendation to publish statistical recommendations for all medical journals so that authors submitting manuscripts can refer to them, e.g. when describing why they used certain statistical tests.

It is noteworthy that fewer than 50% of GBD contributors supported a recommendation to enhance the emphasis on editors seeking statistical reviewers. One proposed solution could involve involving PhD students enrolled in biostatistics courses. These students could earn credit by reviewing multiple articles for statistical accuracy for medical journal editors, supervised by the course instructor. According to a new approach to biostatistics education published in PLOS BIOLOGY, there should be an even greater emphasis on the practical teaching of the subject by, among other things, analyzing articles for statistical validity in topics that correspond to the scope of the studies being undertaken.^23^ Currently, it is very common for many biostatistics curricula to be limited in scope and duration.^24^ Therefore, biostatistics education should emphasize practical applications, such as assisting medical journal editors in reviewing articles where there are concerns about the validity of the conducted analyses. This hands-on approach demonstrates the practical relevance of implementing such recommendations from biostatistics classes.

Of the other most common statistical recommendations identified by GBD contributors, two stand out, namely the organization of short statistical tests for members of editorial boards and online meetings where reviewers and editors would discuss the most important aspects related to the review of the authors' analyses. Dickersin *et al.*, in their publication in JAMA, responded to inquiries regarding the discussions among journal editors when evaluating manuscripts for publication. They emphasized the importance of categorizing editorial discourse as a critical factor in understanding the decision-making process for submitted manuscripts. The authors suggested that comparing the frequencies of negative and positive reviewer comments, as well as examining the language used by editors, would be beneficial in gaining insights into this process.^25^ The presentation to members of editorial boards by reviewers/statistical editors of the most common errors related to the authors' analysis could contribute to improving the quality of published research results in the future. The same applies to the conduct of statistical peer review validation tests, especially in journals that do not have people responsible for this type of activity. Alongside other considerations, it is crucial to emphasize two foundational aspects: the registration of studies prior to their commencement and the development of Statistical Analysis Plans (SAPs). Registration of studies plays a pivotal role in enhancing research transparency and mitigating publication bias. It ensures adherence to pre-specified methodologies, thereby bolstering the reliability and reproducibility of study findings. Similarly, crafting SAPs before data collection begins is essential. These plans delineate rigorous methods for data handling and analysis, guarding against data-driven biases and reinforcing the robustness of statistical inference. Together, effective registration and meticulous SAP development not only promote methodological transparency but also uphold scientific integrity, facilitating critical evaluation and advancing evidence-based practices in research.^26^,^27^ Scientific journals should reject papers that do not comply with appropriate reporting guidelines and should consider rejection if the analysis is not conducted according to a pre-registered plan.

### Strength and limitations

This survey is the first on the opinions of experts, who in this case are GBD collaborators, on the eleven statistical recommendations proposed for implementation in medical journals. To date, the mere publication of such recommendations without their expert evaluation has unfortunately not influenced the level of published research results, nor the frequency with which editors use statistical reviewers. The recommendations analysed were linked to scientific parameters and the respondents' opinions on what percentage of accepted manuscripts they thought were statistically correct. The main objective of the study in this case was not to publish recommendations, as has been the case to date, but to support their implementation in the daily work of medical journals on the basis of the expert opinion obtained. The results of the survey will be sent to a group of more than 3,000 medical journal editors to indicate to them which statistical recommendations, according to scientific experts, should be progressively implemented. In the future, however, there is a need to carry out this type of study on a larger group of researchers from different continents in order to be able to relate the impact of geographical location on the results obtained. In this study, the majority of GBD collaborators were from Asia, Europe and Africa. This is a major limitation of this manuscript, however, as these experts are GBD collaborators, their opinions on the recommendations under study seems valuable. Specifically, research efforts should aim to identify methods that facilitate the gradual implementation of these guidelines. A limitation of this study is that, while the sample aimed to capture a diverse range of academic experiences and publication histories among GBD Collaborators, it may not fully represent the entire spectrum of collaborators, particularly due to the selective nature of survey responses. This may have influenced the generalizability of the findings to the broader GBD community. One more limitation of the research conducted is the remote contact with GBD collaborators. In the future, consideration should be given to conducting this type of research during various scientific events, including those organised by the IHME. These events could facilitate face-to-face interactions, potentially leading to higher response rates and deeper insights into the topics being studied. Future research should be conducted on a larger group of experts, focusing on the most important statistical recommendations outlined in this manuscript. Specifically, the research should aim to identify methods that would gradually facilitate the implementation of these guidelines. The obtained results should be presented, among other venues, at events such as the World Conference on Research Integrity and the International Congress on Peer Review and Scientific Publication. These events serve as pivotal platforms for disseminating findings to a global audience of researchers, policymakers, and stakeholders, thereby fostering informed discussions and promoting the adoption of recommended practices. Additionally, they provide opportunities for critical peer evaluation and validation of the study's methodologies and conclusions, ensuring robustness and credibility within the scientific community. Another limitation of the manuscript is the lower percentage of responses obtained; however, a similar completion rate of surveys was observed in other studies, specifically among members of medical journal editorial boards.^1,15^ Regarding the survey question on the percentage of articles meeting statistical validity in medical journals, it is important to clarify that this inquiry pertained to scientific articles in general and not exclusively to those authored by participants of the study. Participants were asked to provide their opinions based on their collective understanding and experience within the field, aiming to capture a broad perspective on statistical practices across biomedical literature. While acknowledging potential variability in individual responses, the question sought to gather diverse viewpoints to inform broader discussions on statistical rigor in medical research publications.

## Conclusions

In order to enhance the quality of research outcomes in medical journals, it is crucial to prioritize the implementation of statistical recommendations. This involves regular statistical assessments, publication of pertinent guidelines on journal websites, and conducting educational programs for editorial board members.

## Figures and Tables

**Table 1:** Gender, continent of origin and scientific data of GBD collaborators.

Variable		n	%
Continent	Europe	34	23
	Asia	63	42
	Australia	6	4
	North America	12	8
	South America	11	7
	Africa	24	16
Sex	Female	30	20
	Male	120	80
Research internship (years)	1-10	62	41
	11-20	54	36
	21-30	23	15
	>30	11	7
Impact Factor of a journal published GBD article	1-10	35	23
	11-50	33	22
	51-100	31	21
	>100	51	34
Hirsch index	1-10	32	21
	11-50	88	59
	51-100	25	17
	>100	5	3
Number of publications	1-10	5	3
	11-50	52	35
	51-100	32	21
	>100	61	41

**Table 2:** Percentage of GBD fellows supporting statistical recommendations for medical journals.

Statistical recommendations	n	%
Inclusion on the medical journal website of statistical recommendations that authors should consult before considering submitting an article	79	53
Inclusion by authors in the cover letter of information related to the acknowledgement of their reading of the statistical recommendations posted on the journal website	32	21
Informing in the motivation letter about the author(s) responsible for the statistical analysis of the research results obtained	36	24
Inclusion by the authors of a certificate confirming the correctness of the analysis carried out, e.g. a review by an expert biostatistician	42	28
Regular statistical reviews by the editors when there is doubt about the analysis performed by the authors	101	67
Placing more emphasis on editors seeking statistical reviewers for their journals, e.g. PhD students who have completed training in biostatistics and could review several manuscripts as part of their course credit	71	47
Regular publication of biostatistical recommendations in medical journals to educate editorial board members in this area	75	50
Conducting short online meetings for members of editorial boards by biostatistical reviewers/editors to highlight the most common statistical errors made by authors	56	37
Organising meetings for journal editorial boards by the World Association of Medical Editors and the International Committee of Medical Journal Editors to present statistical recommendations	44	29
Organising knowledge tests for members of editorial boards on key aspects related to the validity of statistical analyses carried out by authors	32	21
Publication of uniform for all medical journals of the most important general statistical recommendations to be included in manuscripts submitted by authors	68	45

**Table 3:** The association between the GBD fellows' opinion on the percentage of accepted articles in the medical journals in which the analysis was correctly performed and their Hirsch index, scientific seniority and number of published articles.

Variable		Percentage of accepted articles meeting statistical validity in medical journals								Statistical test result*
		10-30		31-50		51-70		>70		
		n	%	n	%	n	%	n	%	
Hirsch index	1-10	4	13	12	38	8	25	8	25	χ^2^(6) = 5.15; p = 0.53
	11-50	6	7	34	39	25	28	23	26	
	>50	3	10	9	30	5	17	13	43	
Research internship	1-10	7	11	25	40	13	21	17	27	χ^2^(9) = 8.19; p = 0.52
	11-20	2	4	18	33	18	33	16	30	
	21-30	2	9	10	44	5	22	6	26	
	>30	2	18	2	18	2	18	5	46	
Number of articles published in medical journals	1-50	9	16	22	39	15	26	11	19	χ^2^(6) = 11.7; p = 0.07
	51-100	1	3	14	44	9	28	8	25	
	>100	3	5	19	31	14	23	25	41	

*Chi-squared test

## Data Availability

The data presented in this study are available from the corresponding author upon request. The data are not publicly available due to privacy reasons.
